# Combined Relaxation Spectra for the Prediction of Meat Quality: A Case Study on Broiler Breast Fillets with the Wooden Breast Condition

**DOI:** 10.3390/foods13121816

**Published:** 2024-06-09

**Authors:** Bin Pang, Brian Bowker, Seung-Chul Yoon, Yi Yang, Jian Zhang, Changhu Xue, Yaoguang Chang, Jingxin Sun, Hong Zhuang

**Affiliations:** 1 College of Food Science & Engineering, Qingdao Agricultural University, Qingdao 266109, China; bin.pang@qau.edu.cn (B.P.); jxsun20000@163.com (J.S.); 2 College of Food Science & Engineering, Ocean University of China, Qingdao 266003, China; xuech@ouc.edu.cn (C.X.); changyg@ouc.edu.cn (Y.C.); 3 U.S. National Poultry Research Center, USDA-Agricultural Research Service, Athens, GA 30605, USA; brian.bowker@usda.gov (B.B.); seungchul.yoon@usda.gov (S.-C.Y.); 4 School of Computer and Artificial Intelligence, Beijing Technology and Business University, Beijing 100048, China; yangyi@btbu.edu.cn; 5 Institute of Animal Husbandry and Veterinary Medicine, Beijing Academy of Agriculture and Forestry Sciences, Beijing 100097, China; zjcau@126.com

**Keywords:** meat-quality assessment, water-holding capacity, meat texture, NMR, woody breast myopathy, chemometrics

## Abstract

This study evaluated the potential of using combined relaxation (CRelax) spectra within time-domain nuclear magnetic resonance (TD-NMR) measurements to predict meat quality. Broiler fillets affected by different severities of the wooden breast (WB) conditions were used as case-study samples because of the broader ranges of meat-quality variations. Partial least squares regression (PLSR) models were established to predict water-holding capacity (WHC) and meat texture, demonstrating superior CRelax capabilities for predicting meat quality. Additionally, a partial least squares discriminant analysis (PLS-DA) model was developed to predict WB severity based on CRelax spectra. The models exhibited high accuracy in distinguishing normal fillets from those affected by the WB condition and demonstrated competitive performance in classifying WB severity. This research contributes innovative insights into advanced spectroscopic techniques for comprehensive meat-quality evaluation, with implications for enhancing precision in meat applications.

## 1. Introduction

The meat industry continually seeks innovative approaches to enhance the accuracy, precision, and efficiency of meat-quality assessments. In this pursuit, advanced technologies, such as near-infrared spectroscopy (NIRS), computer vision, and hyperspectral imaging (HSI) have emerged as powerful tools for non-intrusive and precise prediction of various meat-quality traits. NIRS utilizes the absorption of near-infrared light by different chemical components in meat to determine its composition. For instance, NIRS can be used to predict the fat, moisture, and protein content, as well as the pH, color, water-holding capacity, and shear force [1]. Computer vision uses image-analysis techniques to extract quantitative information about meat-quality attributes, such as color, meat surface texture, and intra-muscular fat [2]. HSI captures both the spatial and spectral information of meat samples, enabling the prediction of pH, color, texture, and water-holding capacity [3].

On the other hand, time-domain nuclear magnetic resonance (TD-NMR) technology has been a stalwart in investigating meat quality for decades. Studies have demonstrated that water-holding capacity (WHC), meat texture, and chemical composition can be predicted with the water mobility and transverse relaxation time (T_2_) distribution measured by the Carr–Purcell–Meiboom–Gill (CPMG) sequence. The T_2_ distribution spectrum of meat typically consists of three characteristic peaks: hydration water (T_2b_), intra-myofibrillar water (T_21_), and extra-myofibrillar water (T_22_) [4,5]. WHC is closely related to time constants and the corresponding areas of T_21_ and T_22_ [5,6,7], while the meat texture assessed by sensory hardness, juiciness, and tenderness is highly related to the T_2_ distribution spectrum [8,9]. Partial least squares regression (PLSR) models based on the original CPMG spectra were also used to predict beef-quality traits, showing high correlation coefficients (R) for sensory tenderness, Warner–Bratzler shear force, fat content, and moisture content [10]. Despite the success of TD-NMR, challenges arise from the similarities in the longitudinal relaxation time (T_1_) and T_2_ of water and fat molecules, making them insufficient for quantification in food products [11]. To overcome this limitation, Guthausen et al. [12] patented a combined relaxation (CRelax) sequence, enabling the quantification of fat and water by measuring magnetization based on both T_1_ and T_2_. This innovative approach, built on TD-NMR, has proven effective in various applications. Horn et al. [13] employed the CRelax method to quantify the oil and protein contents of cotton seeds, achieving the predictive results with a coefficient of determination (R^2^) of 0.998 for oil content and 0.95 for protein content. Castell-Palou et al. [14] introduced a CRelax method to analyze fat and water contents in cheese, with R^2^ values exceeding 0.97 for both fat and water content. While CRelax spectra provide a powerful tool for food-composition prediction, there is a notable gap in research on using CRelax spectra to predict meat-quality traits, representing an area ripe for further exploration.

The Wooden Breast (WB) condition is an emerging myopathy that affects the pectoralis major in broilers [15]. A distinguishing feature of WB meat is its palpable hardness and rigidness throughout the raw broiler’s breast fillets. The use of broiler breast fillets affected by WB myopathy in a case study is strategic because the meat with different degrees of the WB condition results in a broader range of meat-quality variations. For example, WHC, such as drip loss and cook loss, in the WB fillets is much greater than that in the normal ones [7,16,17,18,19]. In terms of raw-meat texture, compression force [16,20] and shear force [18,21,22] also increase greatly with the severity of the WB condition. Furthermore, WB meat displays an altered chemical composition compared to normal meat. WB meat tends to have higher moisture and fat content but lower protein content [23,24,25,26]. Additionally, the water properties of the WB meat significantly differ from those of normal meat. Tasoniero et al. [20] observed that WB meat had greater T_2b_ and T_21_ relaxation times and their corresponding populations than normal meat. Pang et al. [7] noted that the T_2b_, T_21_, and T_22_ relaxation times and their corresponding relative areas increased with the severity of the WB condition. These differences in meat-quality traits between WB and normal meat provide an ideal scenario to leverage spectra analysis for the development of accurate predictive models.

This study explored the potential of the CRelax sequence within TD-NMR measurements to predict two key meat-quality traits, WHC and meat texture. Using broiler breast fillets (pectoralis major) affected by varying degrees of the WB condition, which results in a comprehensive range of meat-quality traits, the research aimed to demonstrate the applicability of CRelax spectra in predicting variations in meat quality. The results from this case study would provide a potential alternative to subjective scoring of the WB meat based on palpable hardness and rigidity and demonstrate the broader applicability of CRelax spectra in predicting meat quality.

## 2. Materials and Methods

### 2.1. Sample Collection

More than 200 broiler breast fillets (pectoralis major), which were from 8-week-old birds and pre-screened based on palpable hardness and rigidity, were collected from the deboning line of a commercial processing plant at approximately 3 h postmortem. The fillets were trimmed and assigned WB condition scores of normal = 1.0, moderate WB = 2.0, or severe WB = 3.0 according to the criteria used by Bowker et al. [19]. A total of 144 fillets, 48 for each WB condition, were selected for this study.

### 2.2. Water-Holding Capacity Determination

The WHC of the broiler breast fillets was determined by measuring drip loss and cook loss. The fillets were trimmed, dried with paper towels (Wypall, Kimberly-Clark, Irving, TX, USA), weighed at 6 h postmortem, and then stored at 4 °C [27]. The samples were dried again with paper towels and reweighed at 24 h postmortem. Drip loss was calculated as the percentage of weight loss from the initial weight (6 h postmortem). To avoid the influence of texture measurements on cook loss, only 72 out of a total of 144 samples were used for cooking, with the remaining 72 samples used for texture measurements. After reweighing at 24 h postmortem, raw samples were cooked in a Henny Penny MCS-6 combi oven (Henny Penny Corp., Eaton, OH, USA) set at 83.9 °C until they reached a target endpoint temperature of 75 °C. After cooking, the samples were cooled to room temperature, patted dry with paper towels, and reweighed to determine cook loss. The target endpoint temperature of the samples was monitored using thermocouples and a data logger (Model UWTR, Omega Engineering, Stamford, CT, USA) [28]. Cook loss was calculated as the percent changes in weight at 24 h postmortem.

### 2.3. TD-NMR Measurements

TD-NMR measurements were performed on raw intact fillets at approximately 8 h postmortem using an LF90 Proton-NMR analyzer (Bruker Biospin GmbH, Rheinstetten, Germany). Combined relaxation (CRelax) spectra were measured using the CRelax sequence patented by Gauthausen et al. [12], which was provided by Minispec Plus (mq_nf_CRelax_server2, Bruker Biospin GmbH, Rheinstetten, Germany). The fillet temperature was approximately 4 °C during measurements, and TD-NMR measurements were collected at ambient temperature.

### 2.4. Meat-Texture Measurements

The meat texture was evaluated using blunt Meullenet–Owens razor shear (BMORS) [29] shear force as the indicator. The BMORS shear-force measurements followed the procedure described by Zhang et al. [18]. Shear force was measured on raw- or cooked-fillet samples using a Texture Analyzer (Model TA-XT-plus, Texture Technologies Crop, Hamilton, MA, USA) with a 490 N load cell. Seventy-two out of the total 144 fillets were used for raw-fillet BMORS measurements, while the remaining 72 fillets were cooked and used for cooked-fillet BMORS measurements. The razor blade penetrated the fillets perpendicular to the orientation of the muscle fibers at a penetration depth of 20 mm with a test speed of 10 mm/s. Peak shear force and energy were recorded for each fillet. Five BMORS shear measurements were made on each fillet, and the average values of the five peak shear forces and energy were used for data analysis.

### 2.5. Statistical Analysis

One-way ANOVAs of fillet water-holding capacity (drip loss and cook loss) and meat-texture traits (BMORS shear force and BMORS energy of raw or cooked meat) were performed using the PROC GLM procedure of SAS (Version 9.4, SAS Institute Inc., Cary, NC, USA). Significant differences (*p* < 0.05) between three WB conditions (normal, moderate WB, and severe WB) were identified using Tukey’s means separation method.

### 2.6. Multivariate Analysis

To eliminate the effect of sample weight and facilitate the subsequent multivariate analysis, the raw CRelax spectra were subjected to maximum normalization. Following normalization, the normalized CRelax spectra of 144 individual fillet samples (48 for each WB condition) underwent mean-centering and principal component analysis (PCA) based on non-iterative partial alternating least squares (NIPALS) with a maximum of 100 iterations. For the development of the prediction model, partial least square regression (PLSR) was employed to establish quantitative relationships between the normalized CRelax spectra and WHC (drip loss or cook loss) and between the normalized CRelax and meat texture (BMORS shear force or energy). In addition, partial least square discriminant analysis (PLS-DA) was utilized to develop a classification model for distinguishing normal, moderate WB, and severe WB conditions based on the normalized CRelax spectra. To ensure model validity and prevent overfitting, two-thirds of the samples were allocated to a calibration set, and one-third of the samples to a prediction set. The full-cross internal validation was employed to evaluate the model’s performance. The model’s performance was assessed using various metrics, including the coefficients (R) of calibration (Rc), cross-validation (Rcv), and prediction (Rp), as well as the root mean square error (RMSE) of calibration (RMSEc), cross-validation (RMSEcv), and prediction (RMSEp). A desirable model is characterized by high coefficients of determination (Rc, Rcv, and Rp), and low root mean square error (RMSEc, RMSEcv, and RMSEp) [30]. All model development procedures were carried out using Unscrambler X (version 10.3, CAMO, AS, Oslo, Norway).

## 3. Results and Discussion

### 3.1. Meat-Quality Traits of Broiler Breast Fillets with the WB Condition

Table 1 shows the results of water-holding capacity (drip loss and cook loss) and meat-texture traits (BMORS shear force and energy of raw and cooked meat) in broiler breast fillets affected by one of the three WB conditions (normal, moderate WB, or severe WB). Significant differences (*p* < 0.05) were observed in all measured meat-quality traits between the three WB conditions. Compared to normal fillets, WB fillets showed significantly higher values of drip loss, cook loss, and BMORS shear force or energy of raw meat. Notably, only severe WB fillets showed an additional significant increase in BMORS shear force or energy of cooked meat compared to normal fillets. There were no significant differences in BMORS shear force or energy of cooked meat between normal and moderate WB fillets. These data confirm that the WB fillets have a poorer water-holding capacity and greater meat-texture values compared to normal ones, as measured by BMORS, which is consistent with previous reports [17,18,21,22].

### 3.2. Spectrum Description of Broiler Breast Fillets with the WB Condition

**Figure 1:**
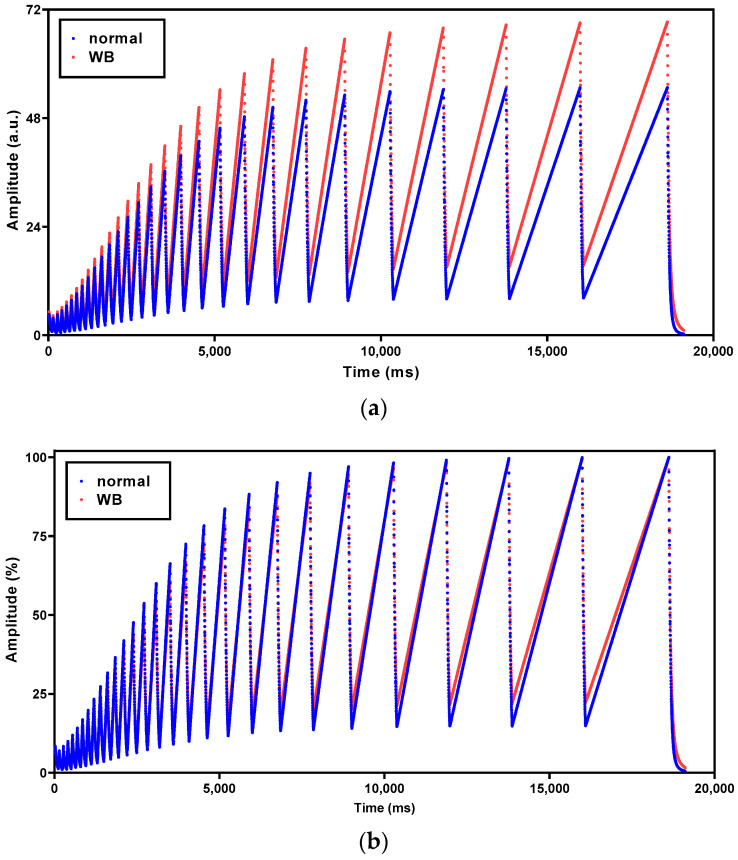
Typical raw (**a**) and normalized CRelax spectra (**b**) after maximum normalization for normal and WB samples.

Figure 1a shows the average raw CRelax spectra of normal and WB samples, exhibiting distinct differences in amplitude across the entire wavelength range (ranging from 21–19,114 ms). Normal samples showed consistently lower amplitudes than WB samples, likely due to the fact that the average WB fillets are typically heavier than average normal fillets [18,25]. To eliminate the effect of sample weight, the raw CRelax spectra were maximum normalized, given that the NMR signal amplitudes are directly proportional to the number of hydrogen nuclei. In the normalized CRelax spectra (Figure 1b), the difference in amplitude, especially in peak valleys, between normal and WB samples was also observed across the entire wavelength range. Previous studies have confirmed that WB meat has longer relaxation times than normal meat [7,20]. This is possibly due to its greater moisture content [23,24,25,26] and more extra-myofibrillar water [20]. The different relaxation behaviors of normal and WB meat can be explained by the different relaxation times of water molecules in different environments. Crystal water exhibits relaxation times of tens of microseconds, bound water has an intermediate relaxation behavior within a few hundred microseconds of NMR signal decay, and free water may show the NMR signal up to seconds [31]. Since the relaxation times of water molecules are strongly dependent on the neighboring surroundings, the greater moisture content and different water component distributions in WB meat may lead to different combined relaxation behaviors.

### 3.3. PCA Analysis

**Figure 2:**
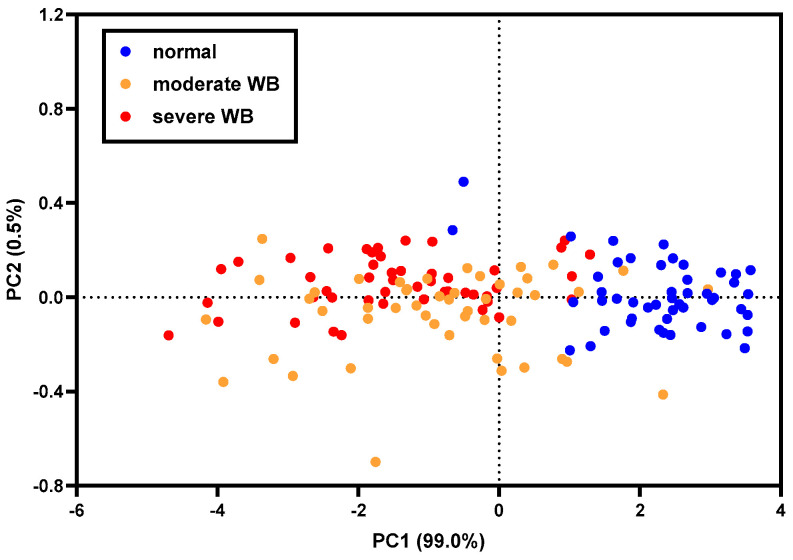
PCA score plots of normalized CRelax spectra for normal, moderate WB, and severe WB samples.

Figure 2 shows the PCA score plots of the first (PC1) and the second (PC2) principal components derived from normalized CRelax spectra. PC1 explained a significant portion of the total variance, accounting for 99.0%, while PC2 contributed an additional 0.5%. Normal fillets were predominantly clustered in the right quadrants of the PCA score plots. In contrast, moderate and severe WB fillets were scattered together, primarily occupying the left quadrants. This pattern underscores the discernible differences between normal fillets and WB fillets. A similar trend was observed in the PCA score plots based on meat water properties (T_2b_, T_21_, and T_22_ time constants and their corresponding relative abundance) [7]. In that report, PC1 explained 80.6% of the total variance, while PC2 accounted for an additional 8.1%. Normal fillets are predominantly grouped in the left quadrants, while moderate and severe WB fillets are mixed and primarily located in the right quadrants. This finding corroborates the results obtained from the CRelax spectra, further emphasizing that the meat characteristics of the normal fillets are distinct from those of the WB fillets.

### 3.4. PLSR Prediction Models for Water-Holding Capacity 

**Figure 3:**
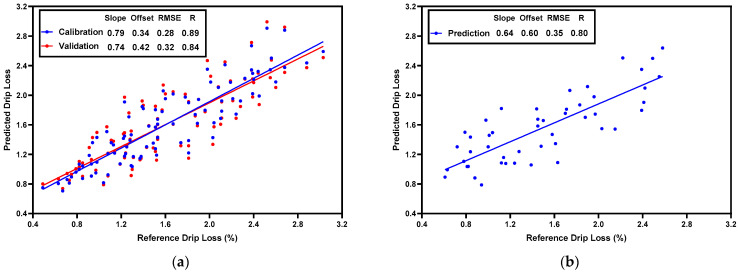
Scatter-plot result of PLSR model for predicting drip loss in the calibration set (**a**) and in the prediction set (**b**).

**Figure 4:**
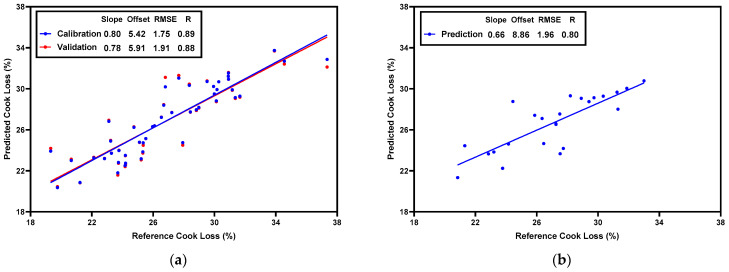
Scatter-plot result of PLSR model for predicting cook loss in the calibration set (**a**) and in the prediction set (**b**).

To predict drip loss, a PLSR model was built based on normalized CRelax spectra (ranging from 21–19,114 ms). The scatter plots of the actual and predicted drip loss values are shown in Figure 3. The model achieved Rc = 0.89, RMSEc = 0.28% in the calibration set, Rcv = 0.84, and RMSEcv = 0.32% in the validation set (Figure 3a), and Rp = 0.80 and RMSEp = 0.35% in the prediction set (Figure 3b). Similarly, to predict cook loss, a PLSR model was built based on normalized CRelax spectra. The scatter plots of the actual and predicted cook-loss values are shown in Figure 4. The model achieved Rc = 0.89, RMSEc = 1.75% in the calibration set, Rcv = 0.88, and RMSEcv = 1.91% in the validation set (Figure 4a), and Rp = 0.80 and RMSEp = 1.96% in the prediction set (Figure 4b). In a previous study, Pang et al. [7] constructed multiple linear regression (MLR) models with meat water properties as the input variables to predict WHC (using drip loss and cook loss as indicators) in broiler breast fillets with the WB condition. The MLR model for drip loss achieved R = 0.79 and RMSEcv = 0.37%, while the MLR model for cook loss achieved an R = 0.87 and RMSEcv = 1.93%. Since meat water properties, such as the time constants of T_2b_, T_21_, and T_22_ and their corresponding relative abundance, were obtained through the CPMG sequences in the TD-NMR measurements and specific regularization algorithms such as CONTIN [32], our results further suggest that the CRelax-based model may have superior WHC prediction capabilities than the CPMG-based model, as evidenced by its higher R and lower RMSE for drip-loss prediction and its comparable R and RMSE for cook-loss prediction.

### 3.5. PLSR Prediction Models for Meat Texture

Texture is an important factor affecting the eating quality of meat. Shear-force measurement is the most common method for measuring meat texture. For broiler breast meat, Meullenet–Owens razor shear (MORS) or blunt MORS (BMORS) shear force and energy have been commonly used to evaluate meat texture [22,29,33,34,35]. Studies have found that BMORS shear force and energy significantly increase with the severity of the WB condition in both raw and cooked fillets [18,21]. Therefore, measurements of the BMORS shear force and the energy of broiler fillets with the different WB conditions were used to build the PLSR prediction models.

**Figure 5:**
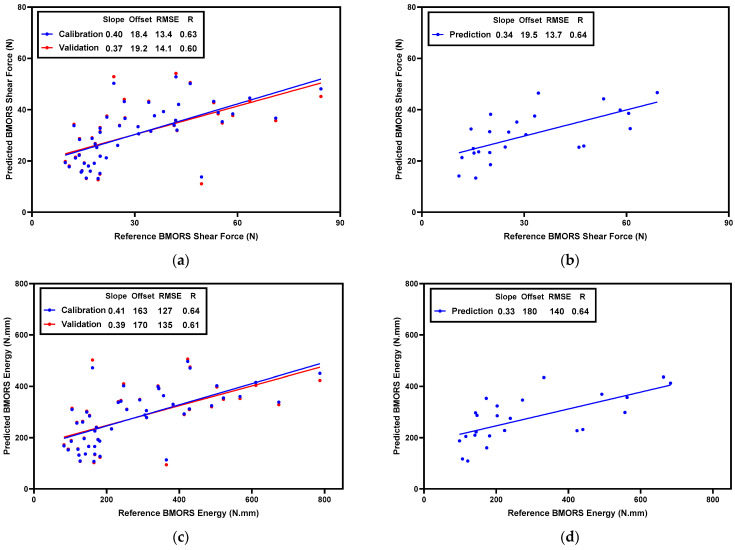
Scatter plots of PLSR models for predicting BMORS shear force and energy in the calibration set (**a**,**c**) and in the prediction set (**b**,**d**) for raw fillets.

To predict the BMORS shear force and energy in raw fillets, two PLSR models were built based on normalized CRelax spectra. The scatter plots of the actual and predicted values are shown in Figure 5. The model for BMORS shear-force prediction in raw fillets achieved Rc = 0.63, RMSEc = 13.4 N in the calibration set, Rcv = 0.60, and RMSEcv = 14.1 N in the cross-validation process (Figure 5a), and Rp = 0.64 and RMSEp = 13.7 N in the prediction set (Figure 5b). For BMORS energy prediction in raw fillets, the model achieved Rc = 0.64, RMSEc = 127 N·mm in the calibration set, Rcv = 0.61, and RMSEcv = 134 N·mm in the cross-validation process (Figure 5c), and Rp = 0.64 and RMSEp = 140 N·mm in the prediction set (Figure 5d). These results suggest that the CRelax-based model has a relatively stable predictive ability across different datasets in raw-meat-texture prediction. 

**Figure 6:**
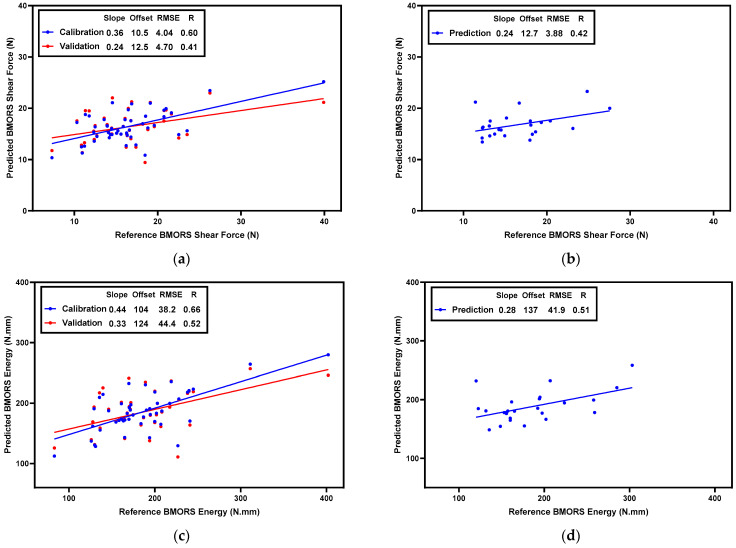
Scatter plots of PLSR models for predicting BMORS shear force and energy in the calibration set (**a**,**c**) and in the prediction set (**b**,**d**) for cooked fillets.

To predict the BMORS shear force and energy in cooked fillets, two PLSR models were built based on normalized CRelax spectra. The scatter plots of the actual and predicted values are shown in Figure 6. The model for BMORS shear-force prediction in cooked fillets achieved Rc = 0.60, RMSEc = 4.04 N in the calibration set, Rcv = 0.41, and RMSEcv = 4.70 N in the cross-validation process (Figure 6a), and Rp = 0.42 and RMSEp = 3.88 N in the prediction set (Figure 6b). For the BMORS energy prediction in cooked fillets, the model achieved Rc = 0.66, RMSEc = 38.2 N·mm in the calibration set, Rcv = 0.52, and RMSEcv = 44.4 N·mm in the cross-validation process (Figure 6c), and Rp = 0.51 and RMSEp = 41.9 N·mm in the prediction set (Figure 6d). These results suggest that, for cooked fillets, the CRelax-based model for the BMORS force prediction has a slightly lower predictive performance than the model for BMORS energy. Furthermore, the predictive performance of the model for cooked fillets is slightly lower than that for raw fillets. This may be because the texture changes in cooked meat are more complex than in raw meat [36], leading to a decrease in the predictive performance of the PLSR model based on CRelax spectra. Overall, the PLSR model based on CRelax spectra might be developed into a promising tool for predicting meat texture and also for predicting the changes in the texture from raw-to-cooked broiler breast meat.

### 3.6. PLS-DA Model for Predicting the Wooden Breast Condition

To predict the WB condition in fillets, a PLS-DA model was developed using normalized CRelax spectra. The model’s performance is summarized in Table 2. The total accuracy of the model for the predicting WB condition was 87.5% for the calibration set and 81.3% for the prediction set. A minor misclassification was observed between the normal and moderate WB samples and between the moderate and severe WB samples; however, no misclassification occurred between the normal and severe WB samples. The model demonstrated excellent performance in identifying the WB fillets, achieving an accuracy of 94.8% in the calibration set and 93.8% in the prediction set. While PCA results (Figure 2) also indicated the ability of CRelax spectra to distinguish the normal and WB meat, the PLS-DA model exhibited superior performance in differentiating the severity of the WB condition.

Previous studies have explored image-processing technology to detect WB myopathy by analyzing physical characteristics, such as fillet air-forced deformation and fillet bending deformation [37,38,39]. However, the technologies are susceptible to errors caused by both the image-acquisition and image-processing methods used to extract specific physical characteristics, as well as, more importantly, the internal meat quality. Near-infrared (NIR) spectroscopy has also been used to distinguish between the normal and WB fillets. Wold et al. [23] developed a linear discriminant analysis (LDA) model based on 3 PLSR components from NIR spectroscopy to identify WB fillets. The model achieved an accuracy of 99.5% in the calibration set and 100% in the prediction set, but it is still not able to distinguish between the moderate and severe WB fillets. Wold et al. [40] further tried to develop LDA models based on principal components (PC) from NIR spectroscopy to identify the WB fillets and classify moderate versus severe WB fillets. The model for the identification of the WB fillets achieved an accuracy of 100% in the calibration set and 94.2% in the prediction set, while the model for the classification of the moderate versus severe WB fillets achieved an accuracy of 76.9% in the calibration set and 68.7% in the prediction set. Our model has a similar performance in identifying WB fillets (with an accuracy of 94.8% in the calibration set and 93.8% in the prediction set), but it performs better for the classification of the moderate versus severe WB fillets. In our model, 55 samples (27 moderate WB and 28 severe WB) out of 64 WB samples in the calibration set were correctly classified (an accuracy of 85.9%), and 25 samples (12 moderate WB and 13 severe WB) out of 32 WB samples in the prediction set were correctly classified (an accuracy of 78.1%). This suggests that the CRelax-based model may have an advantage over the NIR-based model in predicting the severity of the WB condition. Furthermore, a recent study by Pang et al. [17] developed principal component analysis LDA (PCA-LDA) and support vector machine discriminant analysis (SVM-DA) models based on the CPMG spectra. These models exhibited excellent accuracy (100% in the calibration set and 100% in the validation set) in identifying WB fillets, regardless of the mathematical model used.

## 4. Conclusions

In conclusion, this study demonstrated the potential of the CRelax sequence within TD-NMR measurements as a powerful tool for predicting the key meat quality traits. Using broiler breast fillets affected by the wooden breast condition as the case example, the CRelax spectra outperform traditional CPMG spectra in predicting WHC, meat texture, and the severity of WB myopathy. This study contributes valuable insights into the use of advanced spectroscopic techniques for meat-quality assessment, particularly in addressing challenges associated with the emerging myopathy in broiler breast fillets. Future research is warranted to further improve the CRelax spectra models for the prediction of meat-quality traits and explore potential applications of CRelax spectra in diverse meat types and conditions.

## Figures and Tables

**Table 1:** Water-holding capacity and meat-texture traits of broiler breast fillets with the WB condition (mean ± SD).

Trait	The WB Condition		
	Normal	Moderate WB	Severe WB
Drip Loss (%)	1.14 ± 0.53 ^b^	1.64 ± 0.77 ^a^	1.84 ± 0.71 ^a^
Cook Loss (%)	24.1 ± 2.6 ^c^	27.0 ± 2.9 ^b^	30.0 ± 3.1 ^a^
BMORS shear force (raw, N)	17.8 ± 7.7 ^c^	30.3 ± 15.0 ^b^	44.3 ± 17.4 ^a^
BMORS energy (raw, N·mm)	151 ± 57 ^c^	272 ± 142 ^b^	417 ± 179 ^a^
BMORS shear force (cooked, N)	15.0 ± 2.6 ^b^	15.7 ± 4.7 ^ab^	18.6 ± 5.9 ^a^
BMORS energy (cooked, N·mm)	172 ± 24 ^b^	174 ± 49 ^b^	211 ± 62 ^a^

^abc^ Means with different subscripts within a row are significantly different at *p* < 0.05.

**Table 2:** Confusion matrix of PLS-DA classification results by normalized CRelax spectra.

The WB Condition		Actual Result					
		Calibration Set			Prediction Set		
		Normal	Moderate WB	Severe WB	Normal	Moderate WB	Severe WB
Predicted Result	Normal	29	2	0	14	1	0
	Moderate WB	3	28	5	2	12	3
	Severe WB	0	2	27	0	3	13
Accuracy for identifying WB (%)		94.8			93.8		
Total Accuracy (%)		87.5			81.3		

## Data Availability

The original contributions presented in the study are included in the article, further inquiries can be directed to the corresponding author.
